# Supplementary material for: Hyperbolic material enhanced scattering nanoscopy for label-free super-resolution imaging
Source: Nat Commun. 2022 Nov 4;13:6631. doi: 10.1038/s41467-022-34553-6 (PMC9636421; doi:10.1038/s41467-022-34553-6)
Supplement: Supplementary file 1 — Supplementary Information [file 41467_2022_34553_MOESM1_ESM.pdf]

Supporting Information

Hyperbolic material enhanced scattering nanoscopy for label-free super-resolution imaging

Yeon Ui Lee[1,2], Shilong Li[1,3], G. Bimananda M. Wisna[4], Junxiang Zhao[1], Yuan Zeng[4,5], Andrea R. Tao[4,5], Zhaowei Liu[1,4]*

[1]*Department of Electrical and Computer Engineering, University of California, San Diego, 9500 Gilman Drive, La Jolla, California 92093, USA*

[2]*Department of Physics, Chungbuk National University, Cheongju, Chungbuk 28644, South Korea*

[3]*Light-Matter Interactions for Quantum Technologies Unit, Okinawa Institute of Science and Technology Graduate University, Onna, Okinawa 904-0495, Japan*

[4]*Material Science and Engineering Program, University of California, San Diego, 9500 Gilman Drive, La Jolla, California 92093, USA*

[5]*Department of NanoEngineering, University of California, San Diego, 9500 Gilman Drive, La Jolla, California 92093, USA*

*Email address: zhaowei@ucsd.edu

Supplementary Note 1. Organic hyperbolic material (OHM) and tailored scattering

Supplementary Note 2. Numerical simulation of HMES nanoscopy (1)

Supplementary Note 3. Numerical simulation of HMES nanoscopy (2)

Supplementary Note 4. The role of silver nanoparticles

Supplementary Note 5. The role of silver nanoparticle size on the backside of the OHM

Supplementary Note 6. Numerical simulation of HMES nanoscopy (3)

Supplementary Note 7. HMES nanoscopy images of polystyrene beads with different diameters

Supplementary Note 8. Resolution enhancement in different imaging modalities

Supplementary Note 9. The optical transfer function for OHM films with different thicknesses

Supplementary Note 1. Organic hyperbolic material (OHM) and tailored scattering

Three-dimensional (3D) finite difference time domain (FDTD) simulations were performed to investigate the effect of the near-field coupling on the light scattering by the dielectric nanoparticle on the OHM, which shows an enhanced light scattering compared to the case when the same scatterer sitting on a glass substrate, as shown in Figure S1.

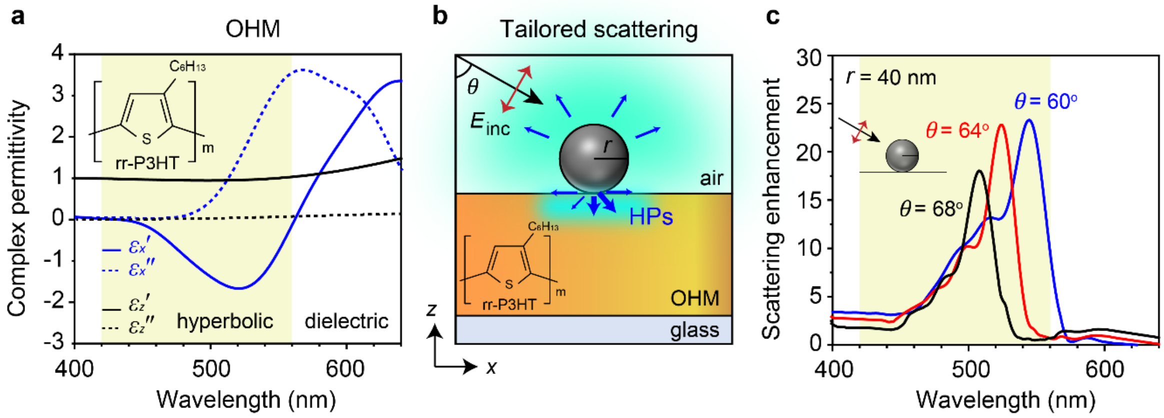

Supplementary Figure S1. Organic hyperbolic material (OHM) of regioregular poly(3-hexylthiophene-2,5-diyl) (rr-P3HT) film in the visible spectral range. (**a**) Chemical structure of rr-P3HT (inset). Horizontal *x*- (blue curves) and vertical *z*-components (black curve) of the real (solid curve) and imaginary (dashed curve) parts of the complex permittivity, ε, of rr-P3HT film; OHM from 420 nm to 560 nm. This OHM film was fabricated and characterized as described in ref. 1. (**b**) Schematic of tailored scattering of a dielectric nanoparticle by OHMs. (**c**) FDTD-calculated scattering enhancement of a dielectric nanoparticle (polystyrene, refractive index $n = 1.6$, radius $r = 40$ nm) situated on the OHM compared to that on the glass, where linearly (p-) polarized laser beam with the polar angle of incidence ($\theta = 60°, 64°, 68°$) was used as the excitation source. The OHM's hyperbolic dispersion range (420 nm < λ < 560 nm) is highlighted.

Supplementary Note 2. Numerical simulation of HMES nanoscopy (1)

Polystyrene nanoparticles situated on top of the OHM are illuminated by a plane wave (total-field scattered-field (TFSF) source at λ = 530 nm, see details in Method section) from polar direction θ = 60° and azimuthal direction ϕ_m (Figures S2a and S2b). The near-field intensity distribution is obtained from monitors M_s in the *xy* planes (blue bars in Figure S2a) and the obtained near-field intensity distributions are shown in Figure S2c. To investigate the practical images obtained from a microscope with a given numerical aperture (NA = 1), the far-field projection of the intensity is performed, where any plane waves with angles outside of the NA are then discarded. After that, the remaining light is re-focused onto an image plane using chirped *z*-transform (Figure S2d) (see Method section for details). The coherently scattered field intensity of the polystyrene nanoparticles situated on top of the OHM illuminated from a different azimuthal direction of the coherent light illumination exhibits typical interferences which make it difficult to detect the dielectric nanoparticles directly. However, by illuminating the dielectric nanoparticles sequentially from all azimuthal directions, the incoherent averaging of the partial scattering images provide a nearly artifact-free image showing the dielectric nanoparticles directly with high contrast (Figure S2d, most right). Note that the recorded

scattering images of the dielectric nanoparticles situated on top of the OHM possess high-k information since the tailored scattering results from the excitation of HPs of the OHM mediated by the dielectric particles.

A region of interest (white box in the inset of Figure S2d) is given in Figure S2e with varying illumination azimuthal directions and with small variations in polar angle (θ = 60°, 62°, 64°). It is important to note that 60 nm silver nanoparticles on the backside of OHM were introduced to provide more various high-k illuminations via small perturbations in the illumination. The small variations in the polar angle of illumination further reduce the spatial coherence at each azimuthal illumination (see Figure S4). As shown in Figure S2e, the centroid of the scattering intensity pattern is slightly shifted in the polar illumination angle.

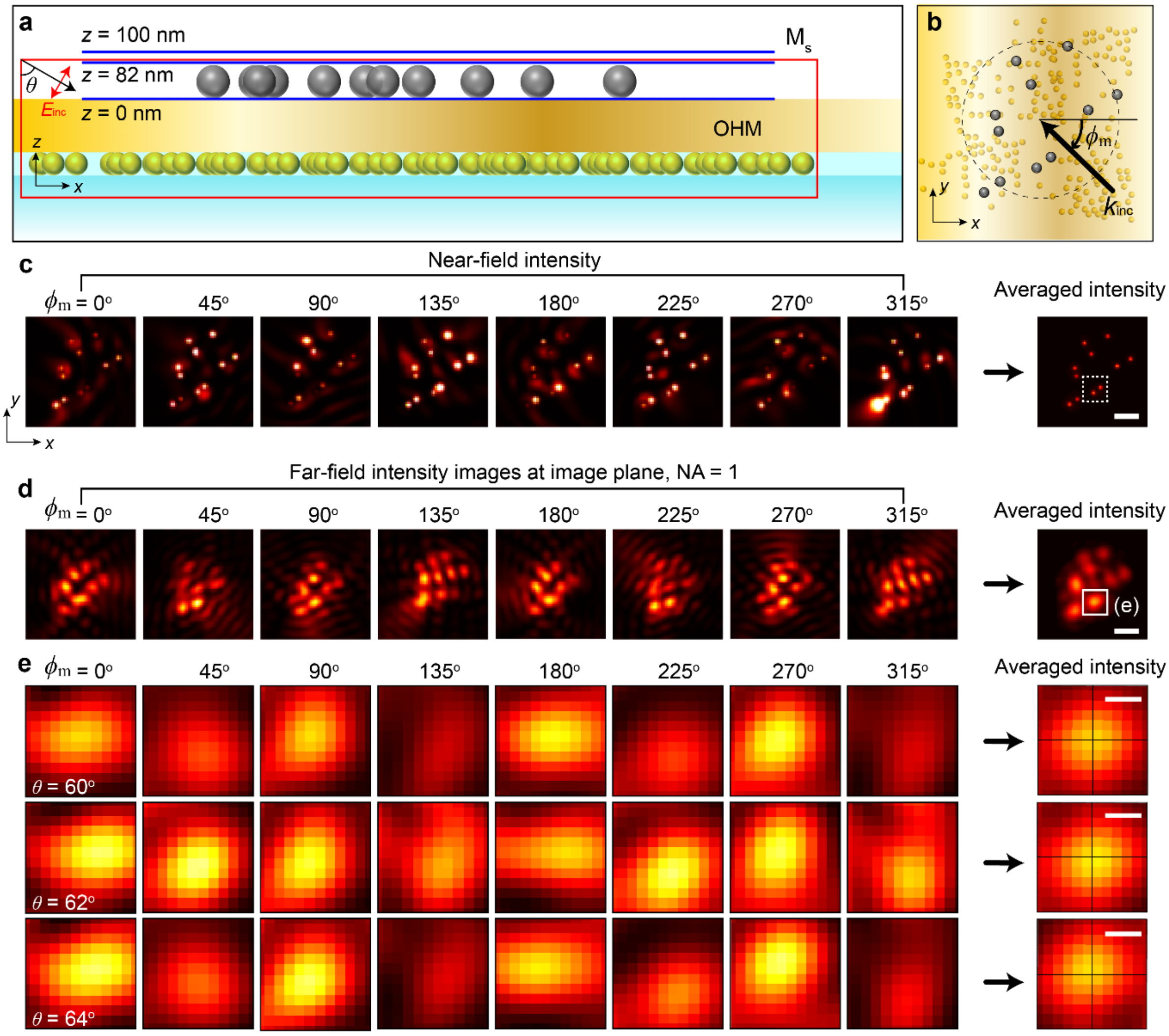

Supplementary Figure S2. Calculated scattering intensity distributions by polystyrene nanoparticles on the OHM. (**a**, **b**) Schematics of FDTD setup with a total-field scattered-field (TFSF) source (red box) used to analyze scattering by polystyrene nanoparticles (r = 40 nm) illuminated by a p-polarized plane wave (E_{inc} at λ = 530 nm, θ = 60°). Silver nanoparticles on the backside of the OHM were introduced to generate various high-k illumination patterns. Three monitors (blue) are placed on the polystyrene nanoparticles to calculate the near-field intensity. (**c**) Near-field intensity distributions in the x-y plane for an azimuthal illumination direction ϕ_m. Inset: Averaged intensity. (**d**) Far-field images calculated from the near-field intensity images with NA = 1. Inset: Averaging

intensity over azimuthal illumination orientations. Scale bar: 300 nm. (**e**) Zoom-in of a region of interest (white box in the inset of (d)) with small variations in polar angle (θ = 60°, 62°, 64°). Scale bar: 100 nm.

Supplementary Note 3. Numerical simulation of HMES nanoscopy (2)

We have further tested the ability of the reconstruction for an object consisting of two curved lines (refractive index n = 1.6) with a 100-nm gap width (Figure S3). Figure S3b shows exemplary results of the partial scattering images. Whereas the gap between two lines cannot be resolved in the averaged partial scattering images (Figure S3c), after the Blind-SIM reconstruction, the two lines are clearly separated (Figure S3d).

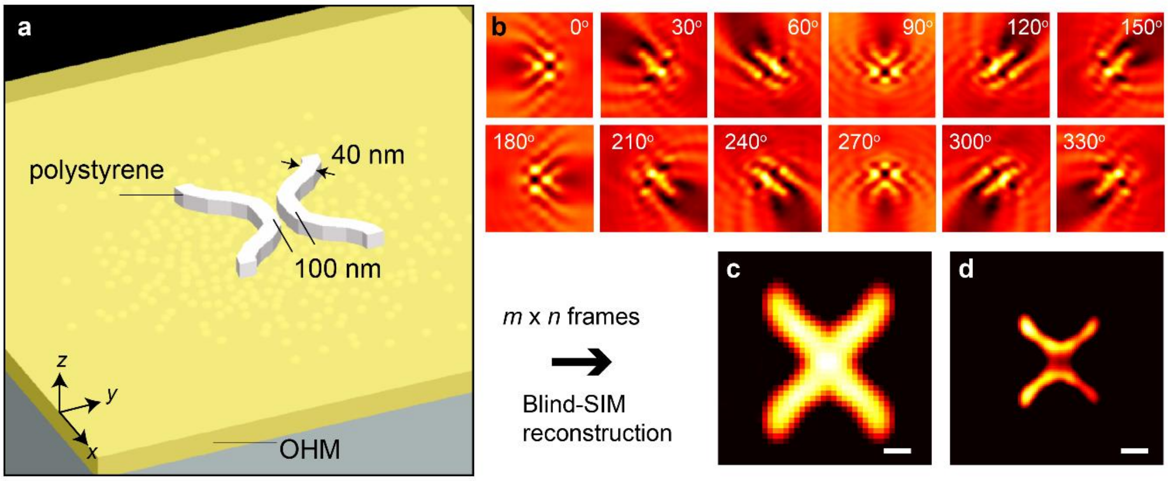

Supplementary Figure S3. Simulated HMES nanoscopy image. (**a**) Schematic of an object consisting of two lines with a 100 nm gap. (**b**) Partial scattering images of the object illuminated from various directions ϕ. (**c**) Averaged image generated from $m \times n$ partial scattering images, where m = 12, n = 10. The objects were illuminated from various directions (ϕ ranging from 0°–330° with an interval of 30° and θ ranging from 60°–69° with an interval of 1°). (**d**) Blind-SIM reconstructed image obtained from the 120 partial scattering images. Scale bar: 100 nm.

Supplementary Note 4. The role of silver nanoparticles on the backside of the OHM

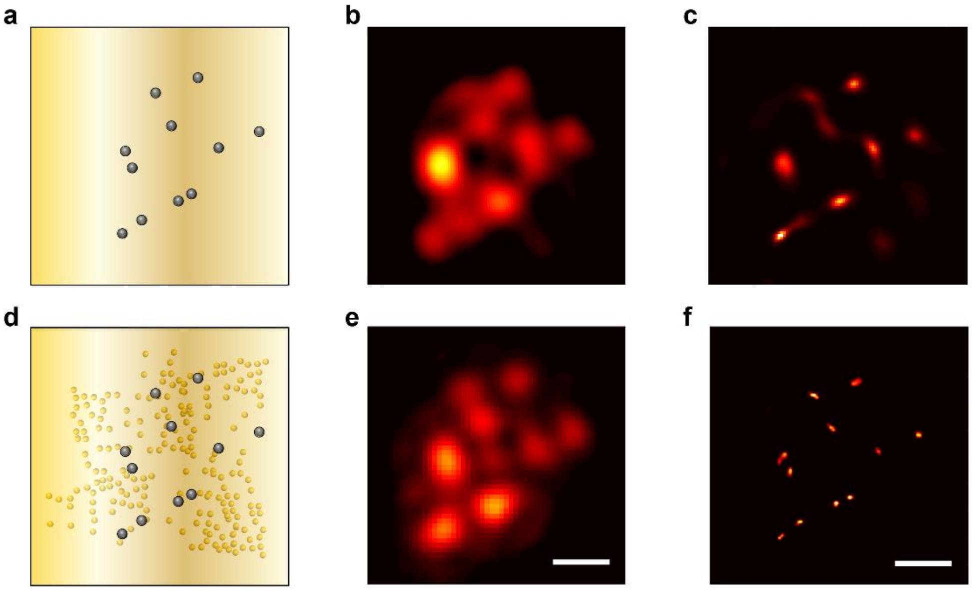

Supplementary Figure S4. The role of silver nanoparticles on the backside of the OHM. (**a**) Polystyrene nanoparticles situated on the OHM. (**b**) Averaged intensity image generated from partial scattering images. (**c**) Blind-SIM reconstructed image. (**d**–**f**) Those in (a–c) when the silver nanoparticles were introduced on the backside of the OHM. Scale bar: 300 nm.

Supplementary Note 5. The role of silver nanoparticle size on the backside of the OHM

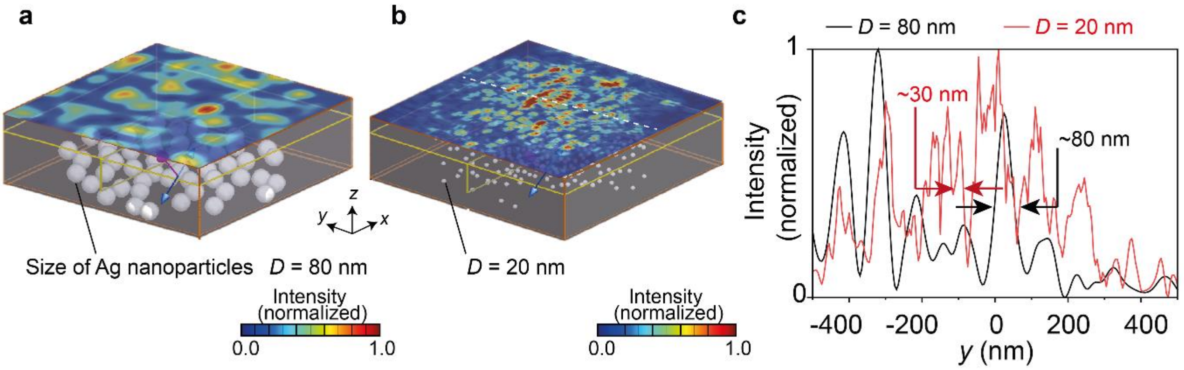

Supplementary Figure S5. The role of silver nanoparticle diameter (*D*) on the backside of the OHM. (**a**, **b**) High-*k* near-field illumination speckles on the sample plane generated by the OHM when the silver nanoparticles with diameters of 80 nm (a) and 20 nm (b) were introduced on the backside of the OHM. (**c**) Cross-section curves.

Supplementary Note 6. Numerical simulation of HMES nanoscopy (3)

The HMES nanoscopy images of adjacent 40-nm polystyrene beads separated from 40 to 80 nm (Figure S6a) are simulated and verified the resolving capability of the HMES nanoscopy. The silver nanoparticles with a diameter of 20 nm on the backside of the OHM were introduced. The corresponding diffraction-limited scattering image is shown in Figure S6b. The

reconstructed super-resolution scattering image based on the HMES nanoscopy is shown in Figure S6c. Two pairs (Figure S6d) and three pairs (Figure S6e) of closely located polystyrene beads are resolved with a distance ~ 40 nm. The Fourier spectrum of Figure S6b (Figure S6c) is shown in Figure S6f (Figure 6g). Figure S6h illustrates the cost function (CF) value for iterations of gradient descent. Figures S6i–S6l show reconstructed images during the operation of the algorithm at the iteration number indicated by the arrows in Figure S6h.

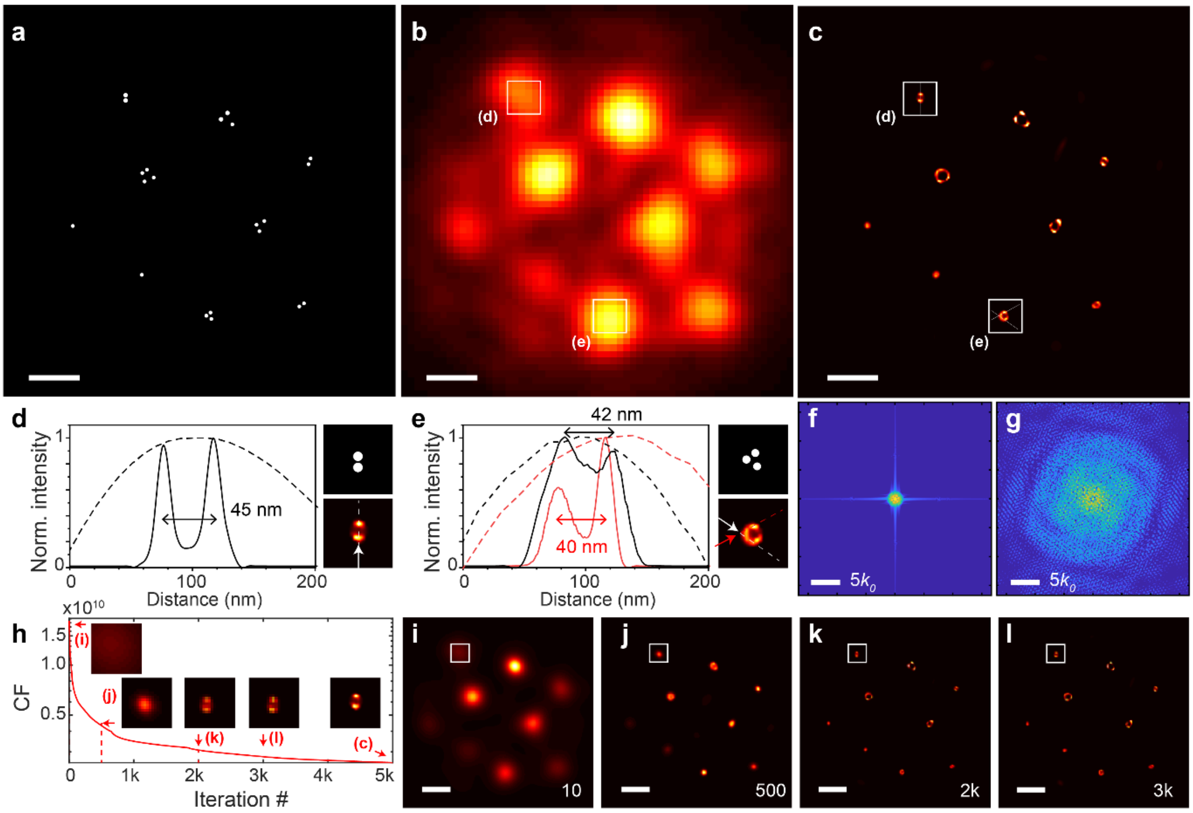

Supplementary Figure S6. Numerical simulation of the HMES nanoscopy images of 40-nm polystyrene beads. (**a**) Imaging object of $r = 20$ nm dielectric nanoparticles. (**b**) A scattering image averaged over 120 images with coherent illumination from various directions. (**c**) Blind-SIM reconstructed image obtained from the 120 partial scattering images. Scale bar: 300 nm. (**d**, **e**) Normalized intensity profiles of mean image (dashed lines, d) and HMES image (solid lines, e) marked with the white rectangles shown in (b, c), respectively. (**f**, **g**) The associated spatial-frequency spectra for the diffraction limited scattering image (b) and HMES image (c), respectively. (**h**) Reduced cost function (CF) versus iterations. (**i**–**l**) HMES images at the number of iteration of 10 (i), 500 (j), 2000 (k), and 3000 (l).

Supplementary Note 7. HMES nanoscopy images of polystyrene beads with different diameters

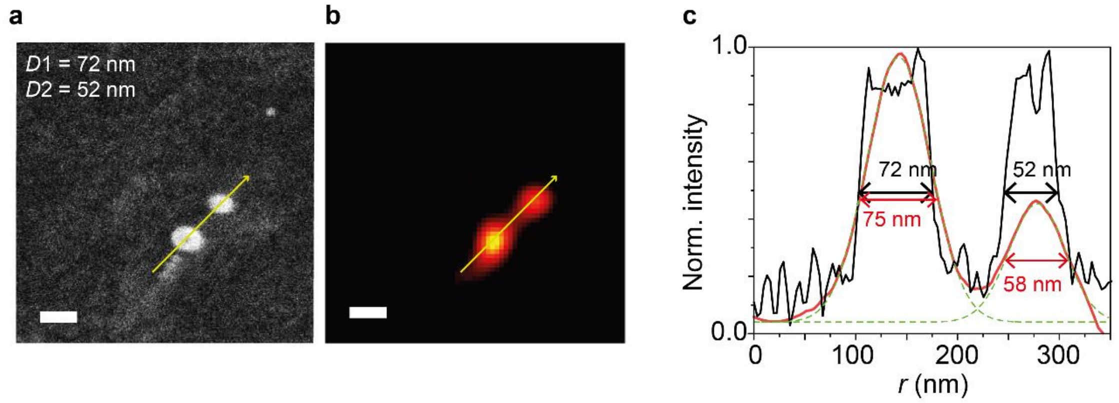

Supplementary Figure S7. HMES nanoscopy images of polystyrene beads with different diameters. (**a**) SEM image of two beads with diameters of *D*1 = 72 nm and *D*2 = 52 nm, respectively. (**b**) Super-resolution label-free scattering images of the polystyrene beads using the HMES nanoscopy. Scale bar: 100 nm. (**c**) Normalized intensity profiles along yellow paths in SEM image (black curve) and HMES image (red curve), respectively.

Supplementary Note 8. Resolution enhancement in different imaging modalities

The fundamental principle of structured illumination microscopy (SIM) revolves around shifting high spatial frequency components of an object into the detectable bandwidth of far-field optics to achieve a larger effective imaging bandwidth. Here we provide a simple theoretical framework, adapted from Refs. 2 and 3, to describe the resolution enhancement via SIM in different imaging scenarios.

Since a fluorescence process responds to the illumination intensity while a scattering process responds to the illumination field, the detected fluorescence intensity $D(r)$ and the scattering intensity $d(r)$ of a sample $s(r)$ are

$$D(r) = [I(r)s(r)]\otimes h(r) \text{ and} \tag{1}$$

$$d(r) = |[E(r)s(r)]\otimes a(r)|^2, \tag{2}$$

in the incoherent imaging and coherent imaging modalities, respectively. Here $I(r)$ is the illumination intensity and $h(r)$ is the incoherent detection point spread function (PSF), while $E(r)$ is the illumination field and $a(r)$ is the coherent detection PSF.

In the case of fluorescence imaging, the optical transfer function (OTF) $\tilde{h}(r)$ has a cutoff frequency at $\tilde{h}_{\text{cutoff}} = 2\text{NA} \times k_0$, where NA is the numerical aperture of the detection optics and k_0 is the free space wavevector at the emission wavelength. For scattering imaging, the coherent transfer function (CTF) $\tilde{a}(r)$ has a cutoff frequency at $\tilde{a}_{\text{cutoff}} = \text{NA} \times k_0$.

For a given illumination field $E_{\text{p}}(r)$ bounded by a maximum spatial frequency k_{p}, the corresponding intensity profile $I_{\text{p}}(r) = |E_{\text{p}}(r)|^2$ is bounded by $2k_{\text{p}}$. It can be readily seen that

the maximum achievable cutoff frequency k_c for fluorescence and scattering SIM are

$$k_{c,fluo} = 2\text{NA} \times k_0 + 2k_p \text{ and} \quad (3)$$

$$k_{c,scat} = \text{NA} \times k_0 + k_p. \quad (4)$$

Since the far-field detection bandwidth cannot be improved further other than using high NA optics, creating illumination pattern with high spatial frequency k_p is the key to improving the resolution of SIM.

With this theoretical framework in place, the resolution enhancement mechanism in different scenarios (I–IV) from different combinations of imaging modalities (coherent or incoherent) and SIM mediums (conventional optics or high-k material) can be clarified below:

I. Scattering imaging using conventional optics. In conventional SIM, a sinusoidal illumination $E_p(r) = \cos(k_p r)$ is generated based on interference of incident beams from two opposite illumination angles, with a maximum wavevector $k_{p,max} = \text{NA} \times k_0$ limited by the illumination optics. However, since the scattering process is linear to the electric field, the collected object information is no more than imaging using oblique illuminations at one angle and then the other (Ref. 2). Both methods achieve an extended spatial frequency bandwidth of $k_{c,scat} = 2\text{NA} \times k_0$ —the Abbe diffraction limit—described by Eq. 4. It is worth noting that, although conventional SIM will not yield any additional information beyond that provided by oblique illuminations in scattering imaging, reconstruction algorithms are still required to obtain the final image from SIM sub-images.

II. Fluorescence imaging using conventional optics. In contrast to the scattering process, a fluorescence process responds to the excitation intensity instead of the field distribution. The interference illumination pattern in fluorescence SIM has an intensity distribution of $I_p(r) = |E_p(r)|^2 = \frac{1}{2} + \frac{1}{2}\cos(2k_p r)$ with $k_{p,max} = \text{NA} \times k_{exc}$, where k_{exc} is the free space wavevector at the excitation wavelength. The improved cutoff resolution is therefore $k_{c,fluo} = 2\text{NA} \times k_0 + 2\text{NA} \times k_{exc}$ as described by Eq. 3. In the case when the Stokes shift of the fluorescence is small, $k_{c,fluo} \approx 4\text{NA} \times k_0$, which corresponds to the commonly acknowledged 2-fold resolution improvement of SIM over the Abbe diffraction limit of $2\text{NA} \times k_0$ (Ref. 3).

III. Fluorescence imaging with high-k material. Illumination patterns with spatial frequency components beyond the traditional diffraction-limit are required to further improve the resolution of SIM. By using materials that support a larger lateral wavevector, such as high refractive index waveguides or hyperbolic metamaterials, the maximum illumination wavevector k_{mat} is limited by the material and can be much larger than that supported by conventional optics. The resulting cutoff frequency is $k_{c,fluo} = 2\text{NA} \times k_0 + 2k_{mat}$, leading to a resolution enhancement of $1 + {}^{k_{mat}}/_{\text{NA} \times k_0}$.

IV. Scattering imaging with high-k material. Although conventional SIM will not yield any

additional information beyond that provided by oblique illuminations in scattering imaging as described in the scenario I, structured illumination with high-k materials in this scenario does lead to an extended imaging bandwidth of $k_{\mathrm{c,scat}} = \mathrm{NA} \times k_0 + k_{\mathrm{mat}}$, with a resolution enhancement of $\frac{1}{2}\left(1 + \frac{k_{\mathrm{mat}}}{\mathrm{NA} \times k_0}\right)$ compared to the Abbe diffraction limit. It is the exceptionally large k_{mat} in the high-k materials that leads to a gain in information compared to the conventional resolution. Reconstruction is requested by the structured illumination to resolve the final super-resolution image.

In brief, the resolution enhancement in different scenarios is summarized in the table below.

	Scattering imaging	Fluorescence imaging
Conventional SIM	I: 1	II: 2
High-k material SIM	IV: $\frac{1}{2}\left(1 + \frac{k_{\mathrm{mat}}}{\mathrm{NA} \times k_0}\right)$	III: $1 + \frac{k_{\mathrm{mat}}}{\mathrm{NA} \times k_0}$

Supplementary Table S1: Resolution enhancement compared to the Abbe diffraction limit of $2\mathrm{NA} \times k_0$ in different scenarios (I–IV) from different combinations of imaging modalities (coherent or incoherent) and SIM mediums (conventional optics or high-k material).

It is now clear that our current work is in the scenario IV where we make use of high-k illuminations supported by low-loss organic hyperbolic metamaterials to enhance the resolution of scattering imaging. To retrieve the super-resolution information, Blind-SIM algorithm is used for image reconstruction. Note that, while Blind-SIM and other similar join-deconvolution algorithms for SIM reconstruction can lead to imaging artefacts when directly applied to coherent scattering images, we mitigate the issue by averaging the scattering sub-frames in the azimuthal direction to generate effective incoherent images of the scatters while retaining all the embedded high spatial frequency information.

The resolution enhancement defined in this theoretical framework allows for a relatively fair comparison among different super-resolution methods in the same imaging modality. To this end, the specific experimental conditions of a super-resolution method, such as the lens NA, the wavelength used, and the applied material system must be provided in addition to the achieved absolute resolution. For example, Table S2 summarizes these specifications of the few state-of-the-art label-free scattering imaging methods, and the corresponding resolution enhancements are thereby derived. As can be seen, compared to the absolute resolution, the resolution enhancement is a more direct and precise parameter to evaluate a super-resolution method. The super-resolution method with a 5.5-fold resolution enhancement demonstrated in our current work has thus pushed the resolution limit of label-free nanoscopies into a new level.

Ref. #	Optical medium	Lens NA, Wavelength, Diffraction-limited resolution	Absolute resolution	Resolution enhancement
Our work	Organic hyperbolic materials	0.6 NA, λ = 532 nm, 443 nm	80 nm	~5.5 fold
4	Scalable photonic chip with grating	1.1 NA, λ = 660 nm, 300 nm	93 nm	~3 fold
5	Fluorescent nanowire ring	0.85 NA, λ = 520 nm, 306 nm	140 nm	~2 fold
6	Fluorescent polymer films	0.85 NA, λ = 532 nm, 313 nm	149 nm	~2 fold
7	Si_3N_4 waveguide	0.95 NA, λ = 488, 561, 647 nm, 257, 295, 341 nm	160 nm	<2 fold

Supplementary Table S2: Resolution enhancement in selected label-free scattering imaging methods.

Supplementary Note 9. The optical transfer function for OHM films with different thicknesses

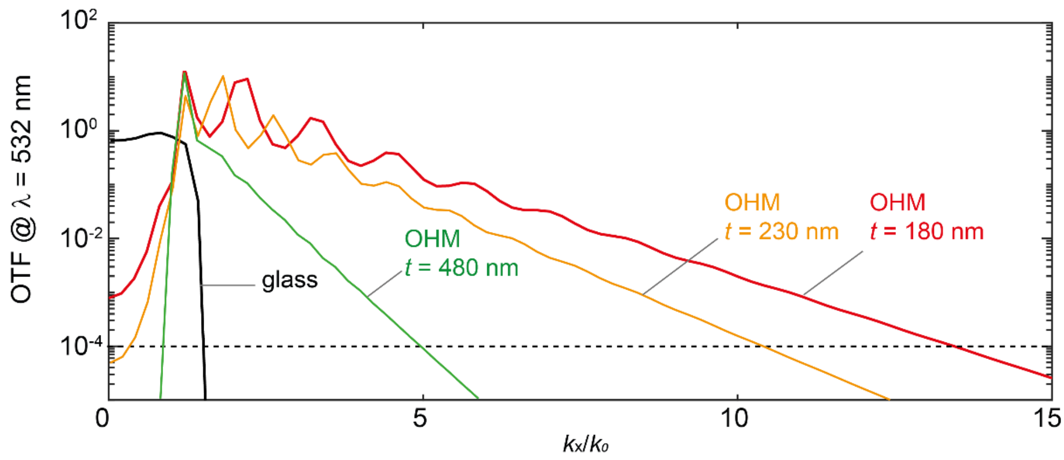

Supplementary Figure S8. Calculated optical transfer function (OTF) in spatial frequency domain of 180-nm, 230-nm, and 480-nm thick OHM films.

References

1. Lee, Y. U., Yim, K., Bopp, S. E., Zhao, J. & Liu, Z. Low-Loss Organic Hyperbolic Materials in the Visible Spectral Range: A Joint Experimental and First-Principles Study. *Adv. Mater.* **32**, 2002387 (2020).

2. Wicker, K. & Heintzmann, R. Resolving a misconception about structured illumination. *Nat. Photonics* **8**, 342–344 (2014).

3. Aminski, C. L. F. K. *et al.* Frontiers in structured illumination microscopy. *Optica* **3**, 667 (2016).

4. Ventalon, C. & Mertz, J. Quasi-confocal fluorescence sectioning with dynamic speckle illumination. *Opt. Lett.* **30**, 3350 (2005).

5. Kim, M. K., Park, C. H., Rodriguez, C., Park, Y. K. & Cho, Y. H. Superresolution imaging with optical fluctuation using speckle patterns illumination. *Sci. Rep.* **5**, 1–10 (2015).

6. Guo, K. *et al.* 13-Fold Resolution Gain Through Turbid Layer Via Translated Unknown Speckle Illumination. *Biomed. Opt. Express* **9**, 260 (2018).

7. Diekmann, R. *et al.* Chip-based wide field-of-view nanoscopy. *Nat. Photonics* **11**, 322–328 (2017).
